# Effect of D-Allethrin Aerosol and Coil to the Mortality of Mosquitoes

**Published:** 2019-09-30

**Authors:** Sayono Sayono, Puji Lestari Mudawamah, Wulandari Meikawati, Didik Sumanto

**Affiliations:** Department of Epidemiology and Tropical Diseases, School of Public Health, Universitas Muhammadiyah Semarang, Semarang, Indonesia

**Keywords:** D-alletrin, Insecticide, *Culex fuscocephala*, *Culex quinquefasciatus*, *Aedes aegypti*

## Abstract

**Background:**

Commercial insecticides were widely used by communities to control the mosquito population in their houses. D-allethrin is one of insecticide ingredients widely distributed in two different concentrations namely 0.15% of aerosol and 0.3% of coil formulations. We aimed to understand the mortality of indoor mosquitoes after being exposed to d-allethrin 0.15% (aerosol) and 0.3% (coil) formulations.

**Methods:**

This quasi-experiment study applied the posttest-only comparison group design. The aerosol and coil d-allethrin were used to expose the wild mosquitoes in twelve dormitory bedrooms of SMKN Jawa Tengah, a vocational high school belonging to Central Java Provincial Government, on March 2017. The compounds were exposed for 60 min to each bedroom with four-week interval for both of formulations. The knockdown mosquitoes were collected into a plastic cup and delivered to the laboratory for 24h holding, morphologically species identification and mortality recording. History of insecticide use in the dormitory was recorded by an interview with one student in each bedroom. Data were statistically analyzed with independent sample t-test and Mann-Whitney.

**Results:**

As many as 57 knockdown mosquitoes belonging to three species were obtained namely *Culex fuscocephala*, *Cx. quinquefasciatus* and *Aedes aegypti* with mortality rate of 50.88% after 24h holding. Knockdown and mortality of mosquitoes were significantly different based on d-allethrin formulations. D-allethrin concentrations were not effective for controlling *Culex* mosquitoes but effective for *Ae. aegypti*.

**Conclusion:**

Further efficacy of d-allethrin 0.15% aerosol to eradicate *Ae. aegypti* is necessary to be conducted in supporting the Dengue vector control.

## Introduction

Mosquitoes play an important role as vector of several kinds of diseases. The different species of mosquito could transmit different disease agents, although some species can transmit a number of pathogens (1, 2). *Aedes* mosquitoes have been known as vector of dengue, Chikungunya and Zika viruses (3) as well as Filarial parasites in certain condition (4). *Culex* mosquitoes can transmit Japanese encephalitis virus and filarial worms, while *Anopheles* transmits malaria and filarial worms. *Armigeres* spp. and *Mansonia* spp. also transmit filarial worms (1, 2).

Some mosquito species are found in residential environments with different abundance. Eight mosquito species were reported from six habitats in Thailand with the order of relative abundance were *Cx. quinquefasciatus*, *Cx. vishnui*, *Cx. gelidus*, *Ae. aegypti*, *Mansonia* spp., *Anopheles* spp., and *Cx. bitaeniorhynchus*, respectively (5). Nine mosquito species were found in urban settlement, in Nigeria namely *Cx. quinquefasciatus*, *Cx. annulioris*, *Anopheles gambiae*, *An. funestus*, *An. rhodesiensis*, *An. arabiensis* and *Ae. aegypti*. Three mosquito species showed the highest relative abundance i.e., *Cx. quinquefasciatus* (50.24%), *Anopheles* spp. (26.5%), and *Ae. aegypti* (0.2%) (6). In Mojokerto, East Java Province, Indonesia, five mosquito species were found in settlements namely *Ae. aegypti*, *Ae. albopictus*, *Ae. laniger*, *Cx. bitaeniorhynchus* and *Cx. quinquefasciatus* (7). *Aedes aegypti* is the dominant species in urban settlements (7) and the Dengue endemic areas (8), while *Cx. quinquefasciatus* is the dominant species in rural area (7).

Urban people in the Dengue endemic areas often used insecticide to repel and against mosquito exposure with several formulations such as repellent, aerosol, mosquito coil and electric mat. The most common of insecticide compounds are d-allethrin, prallethrin, transfluthrin, and diethyl toluamide (DEET) (9, 10). Commercial insecticides usually applied by community with ignored the written instruction (11). This unstandardized practice can cause a negative impact such as mortality of non-target organisms, environmental pollution, and the emergence of insect vectors resistance (12).

The resistance of mosquito vectors to pyrethroid insecticide class has been reported from several countries. *Aedes aegypti* is resistant to several pyrethroid compounds in Vietnam (13), Martinique (14), and Indonesia (15, 16). *Anopheles barbirostris* was resistant to some insecticide compounds namely lambda-cyhalothrin 0.05% and etofenprox 0.5% and tolerant to bendiocarb 0.1% (17). The resistance of *Cx. quinquefasciatus* to pyrethroid was reported from Japan and China that indicated by a genetic mutation (18). This species was reported resistant to permethrin, deltamethrin, and bendiocarb in Benin, Nigeria (19). Permethrin resistant of *Cx. quinquefasciatus* also reported from Central Java, Indonesia (20), and *Mansonia* is tolerant to pyrethroid insecticide (21). *Cx. pipiens* is reported resistant to four of insecticide classes namely pyrethroid, organochlorine, carbamate, and organophosphate in Iran (22).

Household insecticides circulating in Indonesia mostly contain pyrethroid compounds in either single or combination formulations, including d-allethrin. These insecticides are often used in the dense population settlement with uncontrolled doses. Susceptibility of mosquitoes to the compounds is necessary evaluated. We aimed to understand the susceptibility of mosquitoes to d-allethrin 0.15% aerosol and 0.3 % coil compounds in an urban settlement.

## Materials and Methods

### Study site

This quasi-experimental study was conducted in twelve of sixteen bedrooms at the dormitory of SMKN (Vocational High School) Jawa Tengah on March 2017. Each of the 4×6 square meter bedrooms contains six beds and represents the densely populated settlement. The two of d-allethrin formulations were applied at the twelve of bedrooms sequentially.

### Application of d-allethrin aerosol and coil insecticide

**Fig. 1.**
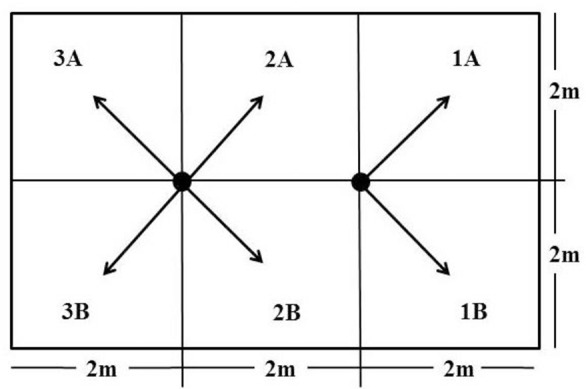
Direction of insecticide spraying. The bedroom was divided into six spraying directions that indicated by the rows

The d-allethrin aerosol insecticide was applied in the morning when the bedrooms are empty. Clothes and snacks were moved out from bedrooms. All of ventilation, windows, door, and holes in the rooms were closed. Temperature and humidity of each room were measured by hygrometer and recorded. Each bedroom was divided into two sections and six quadrant direction for spraying of aerosol insecticide (Fig. 1). Six sprayings were done in each bedroom for five seconds according to the directions based on the previous study (23) with modifications. All of the bedrooms were closed for 60min after the aerosol insecticide was sprayed. The volume of sprayed liquid is equalized by measuring the weight of the insecticide bottle before and after it is sprayed.

The d-allethrin mosquito coil formulation was obtained from the market and applied four weeks following application of the aerosol formulation based on the previous study with modifications (24). A mosquito coil was burned on the tip and placed for 60min in the center of each bedroom. Application of those different insecticide formulations was done in the equal condition of bedrooms.

### Laboratory works

The knockdown mosquitoes from each bedroom were collected in the plastic cups and labeled by name of the bedroom and delivered to Epidemiology Laboratory, Faculty of Public Health of Universitas Muhammadiyah Semarang for twenty-four-hour holding. Each cup was placed in different mosquito cage. Temperature and humidity of the recovery room were maintained at the 27±2 Celsius degree and 80±10%. The ten percent of sugar solution was placed in each mosquito cage for feeding the live mosquitoes. Mortality of mosquito was calculated based on species. Mosquito identification used the arthropod identification key of Walter Reed Biosystematics Unit (25).

### Data analysis

The relative abundance, exposure frequency and dominance rate of mosquitoes were calculated based on the formulas below (26):

$$\text{Relative}\;\text{abundance}=\frac{\text{number}\;\text{of}\;\text{mosquito}\;\text{of}\;\text{a}\;\text{species}}{\text{Total}\;\text{mosquito}\;\text{species}}\times 100\%$$

$$\text{Exposure}\;\text{frequency}=\frac{\text{number}\;\text{of}\;\text{mosquito}\;\text{of}\;\text{a}\;\text{species}}{\text{Total}\;\text{of}\;\text{mosquito}\;\text{catching}}$$

Dominance rate=relative abundance  × frequency

The different of knockdown and mortality of mosquitoes were analyzed based on d-allethrin formulation. History of insecticide use in the dormitory was showed in a frequency table.

### Ethical approval

Protocol of this study was approved by Ethics Committee of Faculty of Medicine of Universitas Diponegoro Semarang. Informed consent and research permission were obtained from dormitory management.

## Results

### Knockdown and mortality of mosquitoes

As many as 57 knockdown mosquitoes were obtained from this study, and mortality of mosquito was 50.88%. Majority of the knockdown (94.44%), dead (96.43%) and alive (92.31%) mosquito were obtained from the d-allethrin aerosol exposed-group, and contrast conditions were found in the mosquito coil exposed-group. A number of the knockdown, dead and alive mosquitoes were significantly different based on the d-allethrin formulations (Table 1).

**Table 1.** Knockdown, dead and alive mosquitoes based on the insecticide (d-allethrin-0.15% compound) formulations

**Insecticide formulation**	**Mosquito conditions**		

	**Knockdown**	**Dead**	**Alive**
**Aerosol**			
**Number**	57	29	28
**Minimum**	1	1	0
**Maximum**	13	7	6
**Mean**	4.50	2.33	2.17
**Standard deviation**	3.26	1.92	1.75
**Mosquito coil**			
**Number**	3	1	2
**Minimum**	0	0	0
**Maximum**	2	1	2
**Mean**	0.25	0.08	0.17
**Standard deviation**	0.62	0.29	0.58
**P-value**	0.01	0.02	0.01

The higher number of mosquitoes was obtained from the aerosol formulation rather than mosquito coil indicating the faster effect of this formulation

### Morphological identification

Results of morphological identification of mosquito were found three species. Two species belong to *Culex* genus and one species belongs to *Aedes* genus, namely *Cx. fuscocephala*, *Cx. quinquefasciatus* and *Ae. aegypti*. Mention order of the three mosquito species also represents the order of relative abundance, exposure frequency and dominance rate (Tables 2, 3).

**Table 2.** Abundance, frequency, and dominance rate of mosquito species

	**Spices**	**Number of mosquitoes**	**Relative abundance (%)**	**Frequency**	**Dominance rate**
	***Culex fuscochepala***	41	71.93	0.45	32.96
	***Culex quinqueasciatus***	15	26.31	0.29	7.67
	***Aedes aegypti***	1	1.75	0.4	0.07

Three mosquitoes species were found in the dormitory with the high abundance of the *Culex* genera

**Table 3.** Knockdown, dead, alive and mortality of mosquitoes based on the species and d-allethrin formulations

**Species**	**Insecticide formulation**	**Knockdown**	**Dead**	**alive**	**Mortality (%)**
***Culex fuscochepala***	Aerosol	41	20	21	48.78
	Mosquito coil	0	0	0	0.00
***Culex quinquefasciatus***	Aerosol	12	7	5	58.33
	Mosquito coil	3	1	2	33.33
***Aedes aegypti***	Aerosol	1	1	0	100.00
	Mosquito coil	0	0	0	0.00
**Total**		57	29	28	50.88

Mortality of mosquitoes after exposed with d-allethrin 0.15% and 0.30% insecticide compounds were very low. This condition indicated that *Culex* mosquitoes were resistant to these concentrations

### History of Commercial Insecticide Use

Results of interview with respondent of each bedroom showed that majority of dormitory inhabitants used insecticide to expel the mosquitoes almost every day, mainly the repellent. The d-allethrin compound was also used among inhabitants (Table 4). Fogging or residual spraying was not used in this dormitory.

**Table 4.** Information about insecticide use among students at the boarding school

**Variables**	**f**	**%**
**Commercial insecticide use**		
**1. Yes**	10	83,3
**2. No**	2	16,7
**Insecticide formulation**		
**1. Aerosol**	1	10,0
**2. Electric mat**	3	30,0
**3. Repellent (lotion)**	6	60,0
**Insecticide compounds**		
**1. Diethyltoluamid 12.5%**	4	40,0
**2. Diethyltoluamid 13%**	1	10,0
**3. Diethyltoluamid 15%**	1	10,0
**4. D-aletrin 0.15% and Praletrin 0.2%**	2	20,0
**5. Praletrin 13.16g/l**	2	20,0
**Frequency of insecticide use in a week**		
**1. 1–3 times**	4	40,0
**2. 4–5 times**	2	20,0
**3. 6–7 times**	4	40,0

Majority of inhabitants at the dormitory use insecticide, mainly in repellent formulation with DEET compound, although the daily use is under 50% of students. Forty percent of inhabitants used pyrethroid compounds

## Discussion

This result showed that d-allethrin aerosol formulation can eradicate and kill more mosquitoes rather than the coil formulation, although this compound indicated effective result for *Aedes* mosquitoes only. This finding required further investigation to obtain sufficient evidence of *Ae. aegypti* susceptibility to d-allethrin. Using the aerosol formulation allowed that the droplets of insecticide will effectively contact with the mosquito’s body, and inhale via respiratory system of mosquitoes (27).

The mortality rate of mosquitoes in this study indicated the low efficacy of d-allethrin 0.15% to the mosquito population in the dormitory rooms. This result matched with the facts that majority of dormitory residents use commercial insecticide for preventing mosquito attack with high frequency, mainly repellant, electric mat, and aerosol formulation. Those insecticide formulations also contain the d-allethrin 0.15% compound (Table 4). High frequency of household insecticide use will correlate with insecticide resistance among *Culex* mosquitoes (28). Resistance of mosquitoes to d-allethrin compounds is similar to the previous report from Malaysia and Indonesia that the mosquito coil containing d-allethrin 0.2% compound resulted in the low mortality rate (24, 29, 30), but different from the reports of aerosol insecticide application containing d-allethrin and d-trans allethrin from Thailand that showed mortality rate of mosquitoes were 96% (31) and 90% (32), respectively. Although distinctly different with the findings in Thailand, results of this study correspond to similar finding in Central Java Province that *Cx. quinquefasciatus* mosquitoes in endemic areas of Lymphatic filariasis have been resistant to the pyrethroid compound, in particular, permethrin 0.75% (20). In Iran *Cx. pipiens* Linn. was resistant to some pyrethroid compounds and other insecticide classes (22). Permethrin and d-allethrin derived from the same class of insecticide, pyrethroid. This insecticide class has target site the voltage-gated sodium channel gene (33). The different types of pyrethroid compounds will have a similar effect to disrupt the voltage-gated sodium channel gene (34) and cause the genetic mutation of codon 1014 of the gene that indicated by substitution of amino acid leucine to phenylalanine or serine (35).

Although the dormitory is located in the Dengue endemic of urban residential, there is only a few the *Aedes* mosquito found. This phenomenon is caused that the dormitory is surrounded by a two-meter high wall fence, and there was no puddle of clean water as a place for microhabitat of this species. This wall can prevent the entrance of mosquito into the dormitory areas. The female *Ae. aegypti* use the opened clean water of the domestic containers in laying their eggs rather than natural water containers such as leaf midrib, tree hole or bamboo stems (36).

*Culex* is the dominant mosquito genus in this settlement. This result is in accordance with the findings in Thailand (5) and Nigeria (6), but different from the findings in Mojokerto, Indonesia where the results of larval surveys in various breeding places in the urban settlement obtained the dominant mosquitoes are *Ae. aegypti* (7). *Aedes aegypti* was the dominant mosquito species in the Dengue endemic areas (8). In this case, the dormitory is located in an urban environment with dense building and surrounded the sewers with stagnant water. This condition provides a good habitat for breeding site of *Culex* mosquitoes. Theoretically, the favorite habitat of this species is stagnant of water surface such as hoof-prints, rain pools, irrigation channels, discarded tubes, ditch, sewer, and ponds (37, 38).

Other studies on the abundance of mosquitoes in the educational environment were reported from Thailand (39), Sabah, Malaysia (40), Makassar, Indonesia (41) that the dominant mosquito is the genus *Culex*, similar to the dormitory of this study although *Cx. quniquefasciatus* is not dominant species (42, 43). The further investigation is needed to analyze the wild mosquito susceptibility to d-allethrin compound by using the laboratory strain of mosquito as the control group, and the causation between environmental profiles, kinds of breeding site and insecticide use to the abundance and dominance of mosquito species, and the occurrence of diseases.

## Conclusion

There were three mosquito species found in the bedrooms of SMKN Jawa Tengah belonging to two genera namely *Culex* and *Aedes*. Exposure of d-allethrin 0.15% in space spray formulation caused higher knockdown and dead mosquito rather than d-allethrin 0.3% in mosquito coil formulation. Overall, the mortality rate of mosquito reached 50.88% which indicated a resistant status to this insecticide compound, except *Ae. aegypti*. Further investigation is needed to determine *Ae. aegypti* abundance at the dormitory and resistant status of *Culex* mosquito to another insecticide compounds.
